# A Moderate Decrease in ADAMTS13 Activity Correlates with the Severity of STEC-HUS

**DOI:** 10.3390/biom13111671

**Published:** 2023-11-20

**Authors:** Khadizha M. Emirova, Olga M. Orlova, Ekaterina M. Chichuga, Alexander L. Muzurov, Piotr P. Avdonin, Pavel V. Avdonin

**Affiliations:** 1 Department of Pediatrics, A.I. Yevdokimov Moscow State University of Medicine and Dentistry, Moscow 127473, Russia; kh.emirova@outlook.com (K.M.E.); olmigor@yandex.ru (O.M.O.); 2 St. Vladimir Children’s City Clinical Hospital, Moscow 107014, Russia; al_muz@mail.ru; 3 Department of Hospital Pediatrics, N.N. Burdenko Voronezh State Medical University, Voronezh 394036, Russia; kagorka@mail.ru; 4 Russian Medical Academy of Continuous Professional Education, Moscow 123995, Russia; 5 Koltsov Institute of Developmental Biology, Moscow 119334, Russia; ppavdonin@gmail.com

**Keywords:** hemolytic uremic syndrome, children, STEC-HUS, ADAMTS13, von Willebrand factor, platelets

## Abstract

Atypical hemolytic uremic syndrome (HUS) develops as a result of damage to the endothelium of microvasculature vessels by Shiga toxin produced by enterohemorrhagic *Escherichia coli* (STEC-HUS). STEC-HUS remains the leading cause of acute kidney injury (AKI) in children aged 6 months to 5 years. The pathomorphological essence of the disease is the development of thrombotic microangiopathy (TMA). One of the key causes of TMA is an imbalance in the ADAMTS13–von Willebrand factor (vWF)–platelet system. The goal of the work was to clarify the role of a moderate decrease in ADAMTS13 activity in the pathogenesis of STEC-HUS. The activity of ADAMTS13 was determined in 138 children (4 months–14.7 years) in the acute period of STEC-HUS and the features of the course of the disease in these patients were analyzed. The study revealed a decrease in the activity and concentration of ADAMTS13 in 79.8% and 90.6% of patients, respectively. Measurements of von Willebrand factor antigen content and the activity of von Willebrand factor in the blood plasma of part of these patients were carried out. In 48.6% and 34.4% of cases, there was an increase in the antigen concentration and the activity of the Willebrand factor, respectively. Thrombocytopenia was diagnosed in 97.8% of children. We have demonstrated that moderately reduced ADAMTS13 activity correlates with the risk of severe manifestations of STEC-HUS in children; the rate of developing multiple organ failure, cerebral disorders, pulmonary edema, and acute kidney injury with the need for dialysis increases. It is assumed that reduction in ADAMTS13 activity may serve as a predictor of disease severity.

## 1. Introduction

Hemolytic uremic syndrome (HUS) in children is one of the types of thrombotic microangiopathy (TMA), which is the most common cause of acute kidney injury (AKI) in children under 5 years of age, with a peak incidence of 6.1 per 100,000/year [1,2,3]. HUS is a clinical and laboratory symptom complex that includes microangiopathic hemolytic anemia (MAHA), thrombocytopenia, and acute kidney injury (AKI). As a result of the developed TMA, partial or complete occlusion of the vessels of the microvasculature occurs with the development of ischemia of the organ, leading to organ dysfunction. The main target organ in HUS is the kidney [4,5].

The most common form of the disease is Shiga toxin-producing *Escherichia coli*-associated HUS (STEC-HUS). Morbidity in STEC-HUS is 85–90%; mortality in the acute period is 2.5–12% due to CNS damage and the development of multiple organ failure syndrome [6,7,8,9]. Extrarenal manifestations occur in about 20% of patients with STEC-HUS, including arterial hypertension, cardiovascular, neurological, gastrointestinal, and other complications that are associated with an increased risk of death [10]. In 25–30% of children after the resolution of STEC-HUS, a decrease in kidney function is detected after a few years, and in 5–15% of cases—arterial hypertension (AH) in 4%—end-stage renal failure [11,12,13]. The development of TMA in STEC-HUS, among other things, is due to a violation of the hemostatic balance towards the activation of thrombus formation processes in conditions of endothelial dysfunction caused by Shiga toxin. At the same time, activation of the plasma coagulation link of hemostasis is secondary. An important role in the development of microthrombosis is attributed to changes in the activity of ADAMTS13 metalloprotease (a disintegrin and metalloprotease with thrombospondin-1-like domains, member 13), which regulates the functional activity of von Willebrand factor (vWF), thereby limiting the growth of blood clots in the microcirculation [14,15,16]. Therefore, the formation of thrombi in the vessels of the microvasculature in STEC-HUS cannot occur without the involvement of the ADAMTS13–vWF–platelet system under the conditions of endotheliotoxic exposure to Shiga toxin. Thus, the study of the state of this system in STEC-HUS is of theoretical and practical interest.

## 2. Characteristics of Patients and Research Methods

### 2.1. Patients

This study included children who met the following criteria: children from 0 months to 18 years in the acute period of STEC-HUS. However, since STEC-HUS is not a common disease, the patients we examined included children with a limited age range from 4 months to 14.7 years who were treated in our clinic.

Diagnosis of the disease was carried out on the basis of an analysis of clinical and laboratory data. The diagnosis of typical HUS was established with the development of Coombs-negative MAGA, thrombocytopenia, and acute kidney injury (AKI) associated with the course of acute intestinal infection (AII).

Etiological verification of AII was carried out by various diagnostic methods. Bacteriological examination of feces was carried out in 138 (100%) children, PCR of EHEC DNA (in fecal samples) in 59 (42.7%) cases, and passive hemagglutination reaction (paired sera) in 27 (19.6%). At the same time, the causative agent of intestinal infection was identified only in 46 (33.3%) cases, of which, in 21 (15.2%) patients—genetic markers of enterohemorrhagic E. coli (EHEC DNA PCR) in 3 (2.1%)—were found specific antibodies to intestinal microorganisms (*Salmonella enteritidis*, *Shigella sonnei*, *Shigella flexneri*) in diagnostically significant titers. Negative results of the study of the etiological component of the infectious process are explained by the use of antibacterial drugs at the prehospital stage, as well as hospitalization of patients in a specialized hospital late in the course of the infectious process and the implementation of STEC-HUS. In patients with STEC-HUS, an increase in the level of leukocytes (Me 18.6 × 10^9^/L [14.5; 23.6]) was detected, associated with AII preceding STEC-HUS.

All children participating in the study were diagnosed with AKI. According to KDIGO Clinical Practice Guideline for Acute Kidney Injury (2012) AKI is definition as any of the following: increase in serum creatinine (SCr) by ≥0.3 mg/dL (≥26.5 μmol/L) within 48 h; increase in SCr to ≥1.5 times baseline, which is known or presumed to have occurred within the prior 7 days; urine volume <0.5 mL/kg/h for 6 h (https://kdigo.org/guidelines/acute-kidney-injury (accessed on 10 November 2023)). The severity of the disease was determined by the development of organ dysfunction. A total of 19 (13.8%) patients were hospitalized in moderate condition, characteriz ed by the classic triad of TMA (MAGA, thrombocytopenia, AKI). A total of 109 (79%) children were hospitalized in a severe condition, where there was a combination of oligoanuric AKI with overhydration and damage to the central nervous system (convulsive syndrome, sopor, stage I coma). The condition was regarded as extremely severe in patients with depression of consciousness to stage II–III coma according to the Glasgow scale, requiring respiratory and cardiotonic support (n = 10; 7.2%). The standard of the classification of the severity of STEC-HUS is presented in Supplement (Table S1).

### 2.2. Measurement of the Activity and Antigen of ADAMTS13 and vWF

Determination of the ADAMTS13 and vWF status was carried out in the acute period of the disease, for which 3 mL of venous blood was taken into a tube with a citrate anticoagulant to obtain plasma. The activity of ADAMTS13 in blood plasma was studied by the FRET (fluorescence resonance energy transfer) method by hydrolysis of the fluorescent substrate FRETS-VWF73 (Peptide Institude, Inc., Osaka, Japan) according to the manufacturer’s protocol. The lower limit of the detection range of ADAMTS13 activity was <5%. The reference limit of ADAMTS13 activity used (according to our study of this parameter in healthy patients) was 80–122%. ADAMTS13 antigen was determined by ELISA using the TECHNOZYM^®^ ADAMTS13 5450551 ELISA kit (Technoclone GmbH, Wien, Austria). Based on published data, a normal ADAMTS13 antigen concentration range of 0.60–1.60 μg/mL was selected.

The activity of vWF in the plasma of patients was determined in the agglutination reaction of lyophilized platelets with ristocetin using a set of reagents and the von Willebrand factor test (Renam, Moscow, Russia, #AG-5). Pooled plasma from healthy donors was used as a standard. The range chosen for vWF activity was 50–150%. The vWF antigen was determined by ELISA using the TECHNOZYM^®^ vWF: Ag 5450201 ELISA kit (Technoclone GmbH, Wien, Austria). The range chosen for the vWF antigen content was 0.5–1.5 U/mL.

## 3. Results

The values of functional activity and concentration of the components of the “ADAMTS13-vWF-platelets” system in patients with typical HUS are presented in Table 1. The median activity of ADAMTS13 was 63.0% [50.8; 78.2]. In most cases (n = 106; 76.8%), metalloprotease activity decreased—it was 58.3% [47.0; 66.0] in thirty (21.7%) patients, determined to be within the reference limits, increasing to 87.0% [84.3; 93.7] only in two (1.5%) cases.

In plasma samples of some patients, we determined the content of the ADAMTS13 antigen, and the activity of vWF and vWF antigen concentration. The median concentration of metalloprotease (ADAMTS13 antigen) was 0.40 μg/mL [95% CI 0.32 to 0.47]. In the vast majority of patients (n = 29; 90.6%), a decrease in the ADAMTS13 antigen (0.36 μg/mL [0.29; 0.47]) was detected, and in only three (9.4%) cases did its concentration correspond to the lower limit of the reference values. According to the correlation analysis, a direct correlation relationship of medium strength was established between the activity of ADAMTS13 and its antigen (r = 0.4266, *p* = 0.0149). We used data from Rieger et al. [17] as reference values. Similar data on the concentration of ADAMTS13 antigen (0.95 ± 0.29 μg/mL) were obtained in the work of Yagi et al. [18].

vWF activity was determined in 64 patients, the Me value of which was 86.2% [14.9; 200.3]. In 26 (40.6%) cases, vWF activity decreased (Me 10.3% [3.0; 21.2]), in 16 (25.0%) cases it corresponded to the norm (Me 181.5% [97.9; 265.8]), and in 22 (34.4%) cases it exceeded the reference values (Me 240.5% [198.9; 327.6]).

The median concentration of vWF (vWF antigen) (n = 47) was 1.4 U/mL [0.9; 2.3]. In six (12.8%) cases, the content of the vWF antigen decreased (Me 0.28 U/mL [0.22; 0.35]), and in other cases it either increased (n = 23; 48.9%)—Me 2.1 U/mL [1.6; 2.9], or it was normal (n = 18; 38.3%)—Me 0.96 U/mL [0.68; 1.07]).

Thrombocytopenia (Me 49.0 × 109/L [38.0; 71.3]) was diagnosed in almost all patients with STEC-HUS (n = 131; 97.8%). Only in 3 patients at the onset of the disease, was a normal platelet level determined, which subsequently decreased. The duration of thrombocytopenia was assessed in 126 (91.3%) patients, the median of which was 11 days [8.8; 14.3].

Taking into account not only the largest number of studies of ADAMTS13 activity, but also the fact that metalloprotease is a natural regulator of thrombosis, the next stage of the work was to conduct a comparative analysis of the severity of the condition of patients with typical HUS, depending on the level of ADAMTS13 activity (n = 138). The patients were divided into two groups; group I included 106 (76.8%) patients with metalloprotease activity below the reference value (<80%), and group II included 32 (23.2%) children with ADAMTS13 activity within the normal range (Me 58, 3% [47; 66] vs. Me 88.0% [84.3; 97.7]; *p* = 0.00000, respectively) (Table 2).

In most patients, the course of AKI was characterized by hyperthermia (n = 118; 85.5%), vomiting (n = 109; 78.9%), diarrhea (n = 136; 98.5%) with hemocolitis (n = 90; 65.2%). In 52 (37.7%) patients, TMA developed on days 2–3 of AII, in half of the cases (n = 69; 50%) on days 4–6, and at a later date (from 7 to 14 days) in 17 (12.3%) children. A total of 118 (85.5%) children required RRT (renal replacement therapy). When analyzing clinical data, anuria was detected more often in children with reduced ADAMTS13 activity compared to patients with normal metalloprotease values (OR 6.1; 95% CI 2.42–15.31; *p* < 0.0001). A decrease in ADAMTS13 activity <80% increases the relative risk of developing anuria by 1.58 times (95% CI 1.1528 to 2.156; *p* < 0.0044). The duration of anuria did not differ between the groups. With ADAMTS13 activity ≥80% (group II), the frequency of oliguria was significantly higher compared with children from group I (11.3% vs. 40.6%; *p* = 0.00046). In an acute episode of TMA, cortical necrosis was detected by ultrasound 1.7 times more often among patients with metalloprotease activity <80% (Table 2).

The severity of the condition of patients with typical HUS was due to the multisystem nature of the lesion. In 63% of cases, signs of volume overload were diagnosed, multiple organ failure syndrome (MOFS) developed in 66 patients (47.8%), while 26 (39.4%) children had dysfunction of two systems, and 40 (60.6%) children had dysfunction of more than two systems. MOF was diagnosed 1.7 times more often in group I (52.8% vs. 31.2%; *p* = 0.02). ADAMTS13 activity below 80% significantly correlated with the development of MOF (OR 2.46; 95% CI 1.0646 to 5.7028; *p* = 0.035).

The spectrum of extrarenal complications was similar in the groups. However, among patients with ADAMTS13 activity below 80% (group I), central nervous system damage was diagnosed significantly more often (50.0% vs. 21.9%; *p* = 0.0075), and pulmonary edema developed 4.9 times more often (30.2% vs. 6.2%; *p* = 0.0047). A decrease in metalloprotease activity <80% significantly correlated with central nervous system damage (OR 3.57; 95% CI 1.42–8.97; *p* = 0.0067) and the development of pulmonary edema (OR 6.49; 95% CI 1.46–28.8; *p* = 0.014). The incidence of hypertension and cardiac damage did not differ between groups. In both groups, signs of gastrointestinal lesions were observed with almost the same frequency (Table 2). Among children of group I, in 1/3 of cases, the condition was complicated by the development of sepsis in response to an infectious process (33.0% vs. 18.7%; *p* > 0.05), which contributed to the aggravation of the severity of STEC-HUS.

In the acute period of the disease, three cases of death were recorded, in which ADAMTS13 activity was reduced.

Using ROC analysis, we determined ADAMTS13 activity thresholds that could better predict severe complications in patients with STEC-HUS (Table 3). The threshold values were 10–20% lower than the limit we set at 80%; however, the conclusions given above and obtained from the ROC analysis were consistent with each other. Using ROC analysis, we identified ADAMTS13 activity thresholds that may better predict severe complications in patients with STEC-HUS (Table 3). The threshold values were slightly lower than the limit we set at 80%; however, the conclusions given above and that obtained from the ROC analysis were consistent with each other. According to the ROC analysis, a decrease in ADAMTS13 activity correlates with (has prognostic power in relation to) anuria, pulmonary edema, MOF syndrome, CNS lesion. In three patients who died, ADAMTS13 activity was below 59%. According to ROC analysis, there is no correlation between decreased ADAMTS13 activity and heart failure, GIT lesion, and arterial hypertension in patients with STEC-HUS.

The laboratory parameters in groups I and II were assessed (Table 4). The severity of anemia, thrombocytopenia and its duration, azotemia, hyperbilirubinemia, increased LDH, SFMC, TV, and fibrinogen did not differ statistically significantly within groups I and II.

Patients with typical HUS with metalloprotease deficiency were characterized by more pronounced leukocytosis (Me 19.5 × 109/L [14.5; 25.9] vs. Me 16.5 × 109/L [14.0; 19.6]; *p* = 0.04), increased CRP (Me 14.8 mg/L [0.9; 48.9] vs. 8.2 mg/L [1.0; 14.6]; *p* > 0.05), and consumption of C3 complement (Me 76.0 mg/dL [70.0; 87.5] vs. 97.0 mg/dL [79.0; 105.0]; *p* = 0.0001), which proves the activation of the alternative complement pathway in this form of the disease within the current infectious process. A correlation analysis revealed an inverse correlation of ADAMTS13 activity with the severity of leukocytosis (R = −0.3, *p* = 0.002) and direct correlation with C3 concentration (R = 0.5101, *p* < 0.0001).

Proteinuria in the acute period of the disease was characteristic of almost all patients, regardless of the level of metalloprotease activity. At discharge from hospital, proteinuria as a marker of kidney damage was more often recorded in patients with deficiency of metalloprotease activity (81.5% vs. 66.7%; *p* > 0.05).

When assessing the state of hemostasis, the level of fibrinogen and the duration of thrombin time corresponded to the reference values. Increases in D-dimers and SFMC were characteristic of children with STEC-HUS, regardless of the level of metalloprotease activity. In this case, an increase in the level of SFMC is associated both with the expansion/stratification of the fibrinogen pool and with the deterioration of the normal polymerization of fibrin monomers. The multiple increases in D-dimer concentration reflect the activation of intravascular hemocoagulation and fibrinolysis. The data obtained indicate that the process of occlusive thrombus formation in typical HUS does not occur without activation of the hemostatic system.

The need for plasma replacement therapy (RRT) and its duration was significantly higher in children with reduced enzyme activity (91.5% vs. 65.6%; *p* = 0.0008; and 22 [11.0; 36.0] days vs. 13 [5.5; 23] days; *p* = 0.0003, respectively), which is confirmed by the results of correlation analysis in the form of feedback of the average strength of ADAMTS13 activity from the need for dialysis (r_pb_ = −0.3, *p* = 0.0002). Decrease in ADAMTS13 activity <80% highly significantly correlated with the risk of applying RRT (OR 5.65; 95% CI 2.08–15.34; *p* = 0.0008).

## 4. Discussion

In this work, we showed that the content and activity of ADAMTS13 is reduced in children with STEC-HUS. A moderate correlation was found between these indicators. The revealed decrease in the activity and concentration of ADAMTS13 can be explained by the consumption of metalloprotease as a result of its binding to an excess of UL-vWF formed under conditions of endothelial damage in typical HUS. Most studies provide evidence that ADAMTS13 activity may decrease only in a few cases in patients with HUS [19,20,21,22,23]. Our data are consistent with the results of a comparative study by N.A. Khalifa et al. [24], which demonstrated a decrease in ADAMTS13 activity in all children with HUS compared with healthy patients. Similar changes in the activity and concentration of ADAMTS13 were found in sepsis [16,25], cardiovascular diseases [26], and COVID-19 [27,28,29].

Thus, in patients with STEC-HUS, there are characteristic changes in the ADAMTS13–vWF–platelet system. The interaction between the three components of this system is a multi-stage process. In the development of typical HUS, the endothelial thromboresistant phenotype is lost as a result of exposure to Shiga toxin, which leads to the release of large amounts of vWF from damaged endothelial cells.

It should be noted that the expression of ultra-large vWF multimers, which have the highest prothrombogenic activity, can be explained not only by a deficiency in ADAMTS13 activity, but also by a violation of their clearance in the areas of occlusion of microvasculature vessels in STEC-HUS [30]. An imbalance between ADAMTS13 and vWF triggers the exposure of thrombogenic components in the intima to coagulation factors and platelets, leading to their activation and aggregation, which supports microthrombosis with the development of target organ ischemia.

Our goal was to find out whether there is a relationship between the state of the ADAMTS13–vWF–platelet system and the severity of STEC-HUS. Most attention has been paid to ADAMTS13, since the determination of the activity of this enzyme has become a routine and fairly accurate test. A decrease in ADAMTS13 activity below 5–10% is the cause and reliable indicator of Upshow–Shulman syndrome and autoimmune thrombotic thrombocytopenic purpura, while the role of a moderate decrease in ADAMTS13 activity in other forms of thrombotic microangiopathy has been studied to a lesser extent. Previously, Khalifa et al. [24] showed significant negative correlations between ADAMTS-13 level in children with STEC-HUS and duration on dialysis. Our analysis of the ADAMTS13 state in 138 children with STEC-HUS showed that signs of a severe condition such as anuria, MOF, CNS lesion, and pulmonary edema are significantly correlated with a reduced level of activity of this enzyme.

It is of interest to analyze cases of complete absence of ADAMTS13 activity in patients with hemolytic uremic syndrome. Cases of STEC-HUS or aHUS patients who simultaneously have ADAMTS13 deficiency are very rare. Hunt et al. [19] described the condition of an 18-month-old patient with diarrhea-associated hemolytic uremic syndrome with almost zero ADAMTS13 activity. The child had anuria and neurological disorders, manifested as a fluctuating level of consciousness. The reason for the decrease in ADAMTS13 activity has not been established. Subsequently, at the age of 5 years, it was normal. This result is close to our data on CNS disorders in patients with reduced ADAMTS13 activity. A similar case of a decrease in ADAMTS13 activity to a level of less than 5% with no detectable inhibitor was described in a 4-year-old girl who had STEC-HUS [22]. ADAMTS13 activity in her blood plasma completely returned to a normal level in remission. At 2-year follow-up, the child had normal blood pressure, serum creatinine 52 μmol/L, no proteinuria, with microalbuminuria level of 27 mg/L. In contrast, 20 STEC-HUS patients with activities between 50 and 100% after a 3-month remission all kept ADAMTS13 activity level similar to that of the acute phase [22]. Data regarding the course of STEC-HUS in this group of patients are not presented in the paper. In patients with atypical HUS with congenital deficiency of ADAMTS13 (Upshaw–Schulman syndrome), the disease occurs in a severe form; it starts at birth with jaundice, distress, severe hemolytic anemia, and thrombocytopenia. Subsequently, kidney and brain damage progresses in these patients [22]. However, Nakayama et al. [31] described a case of STEC-HUS in a 21-year-old woman with ADAMTS13 activity <10% during the acute phase, who had no neurological symptoms. In the paper of Remuzzi et al. [21], five cases of adult patients with recurrent and familial HUS who had complete deficiency of ADAMTS13 activity are presented. These patients could not be clinically distinguished from those with normal ADAMTS13 activity in terms of incidence of end-stage renal disease and neurologic symptoms. Inhibitory antibodies were not detected in any of these patients. In all of them, the deficiency of ADAMTS13 activity remained in remission, which suggests its genetic cause. It can be assumed that in children, unlike adults, even a moderate decrease in ADAMTS13 activity is a pathogenetic factor in the development of severe complications in STEC-HUS. The more severe course of this disease in children may be due to the synergistic effect of decreased ADAMTS13 activity and complement activation [32].

In our study, for the first time, using significant clinical material, a comprehensive assessment of the state of the “ADAMTS13–vWF–platelet” system in children with STEC-HUS was carried out. The vast majority of patients showed a decrease in the activity and antigen of ADAMTS13. We have demonstrated for the first time that a moderate decrease in ADAMTS13 activity correlates with central nervous system disorders, MOF syndrome, anuria, and pulmonary edema in STEC-HUS. Determination of ADAMTS13 activity has become an almost routine test that is performed in patients with suspected HUS upon admission to the clinic to exclude thrombotic thrombocytopenic purpura. Plasma must be collected as early as possible from the manifestation of the disease and strictly before plasma therapy, which can be considered a limitation of the study of ADAMTS13 activity in STEC-HUS. Moderately reduced activity of this enzyme can be considered in combination with other clinical findings as an indicator of subsequent severe manifestations of STEC-HUS. However, determination of ADAMTS13 activity can be used for prognostic purposes in assessing the severity of STEC-HUS, but not as an indicator of the disease itself. At the same time, the advantages of this test include the routineness and simplicity of the study and the small volume of plasma required, which becomes significant in conditions of intensive care and anemia accompanying HUS. This will allow planning in advance such a measure as plasma therapy (fresh frozen plasma infusion or plasma exchange), which is considered in children with STEC-HUS with neurological symptoms [33].

## Figures and Tables

**Table 1:** State of the ADAMTS13–vWF–platelet system in STEC-HUS.

	n (%)	Reference Range	Me	IQR	%		
					↓	N	↑
ADAMTS13 activity, %	138 (100%)	80–122 *	63.0	50.8; 78.2	76.8	21.7	1.5
ADAMTS13 antigen, µg/mL	32 (23.2%)	0.74–1.42 **	0.40	0.3; 0.5	90.6	9.4	-
vWF activity, %	64 (46.4%)	50–150	86.2	14.9; 200.3	40.6	25.0	34.4
vWF antigen, U/mL	47 (34.1%)	0.5–1.5	1.4	0.9; 2.3	12.8	38.3	48.9
Platelets, ×10^9^/L	134 (97.1%)	180–320	49.0	38.0; 71.3	97.8	2.2	-

n—number of patients, Me—median, IQR [25; 75]—interquartile range, N—within reference values, ADAMTS13—a disintegrin and metalloprotease with thrombospondin-1-like domains, member 13, vWF—von Willebrand factor. * The reference range for ADAMTS13 activity is based on our data on the activity of this enzyme in healthy individuals. ** The reference range for ADAMTS13 antigen was taken from [17].

**Table 2:** Clinical characteristics of patients with STEC-HUS depending on ADAMTS13 activity.

INDEX	n	Group I ADAMTS13 < 80%	n	Group II ADAMTS13 ≥ 80%	*p*
ADAMTS13, %; Me [25; 75]	106	58.3 [47.0; 66.0]	32	88 [84.3; 97.7]	0.00000
Diuresis	106		32		
anuria	94	88.6%	18	56.2%	0.00014
oliguria	12	11.3%	13	40.6%	0.00046
saved	0	0.0%	1	3.2%	
Duration of anuria, days; Me [25; 75]	94	8.0 [5.8; 13.0]	18	8.5 [5.0; 12.5]	0.88
<7 days	40	42.5%	7	38.9%	
>7 days	54	57.5%	11	61.1%	
Cortical necrosis	22	20.7	4	12.5%	0.44
Hemorrhagic syndrome	83	78.3%	23	71.9%	0.48
MOF syndrome	56	52.8%	10	31.2%	0.043
CNS lesion	53	50.0%	7	21.9%	0.0075
Heart failure	38	35.8%	9	28.1%	0.52
Pulmonary edema	32	30.2%	2	6.2%	0.0047
GIT lesion	42	37.3%	9	28.1%	0.30
Arterial hypertension	82	77.3%	24	75.0%	0.81
Sepsis	35	33.0%	6	18.7%	0.18
Death	3	2.8%	0	0.0%	1.00

Note: n—number of patients, Me [25; 75]—median and interquartile range, *p*—statistical significance of differences, MOF—multiple organ failure syndrome, CNS—central nervous system, GIT—gastrointestinal tract. *p* was calculated according to Fisher’s exact test.

**Table 3:** ROC analysis for ADAMTS13 activity (%) associated with the severity of STEC-HUS.

INDEX	AUC	*p*	Threshold, %	Sensitivity, %	Specificity, %
Anuria	0.752 [0.671; 0.822]	<0.0001	≤70.6	71.43	73.08
MOF syndrome	0.612 [0.525; 0.694]	0.0195	<62	59.09	59.72
CNS lesion	0.645 [0.559; 0.725]	0.0021	≤76	86.67	35.90
Pulmonary edema	0.666 [0.580; 0.744]	0.0025	≤59	64.71	66.35
Heart failure	0.556 [0.469; 0.640]	0.2804	≤61	59.57	60.44
Arterial hypertension	0.519 [0.432; 0.605]	0.7579	≤34.8	3.77	87.50
GIT lesion	0.565 [0.478; 0.649]	0.1902	≤64	68.63	54.02
Death	0.832 [0.756; 0.892]	0.0017	≤59	100.00	62.20

AUC—area under curve.

**Table 4:** Laboratory characteristics of patients with STEC-HUS depending on ADAMTS13 activity.

Index	n	Group I ADAMTS13 < 80% Me [25; 75]	n	Group II ADAMTS13 ≥ 80% Me [25; 75]	*p*
Hemoglobin, g/L	106	65.0 [56.0; 73.0]	32	64.5 [61.0; 74.5]	0.48
Platelets, ×109/L	106	48.0 [38.0; 69.0]	32	49.0 [39.0; 78.0]	0.42
Duration of thrombocytopenia, days	96	11.0 [9.0; 15.0]	30	11.0 [7.8; 12.3]	0.34
<10 days	46	47.9%	14	46.7%	0.59
≥10 days	50	52.1%	16	53.3%	0.59
Leukocytes, ×109/L	100	19.5 [14.5; 25.9]	30	16.5 [14; 19.6]	0.04
Creatinine, µmol/L	106	453.6 [338.0; 570.1]	32	400.4 [171.1; 596.5]	0.18
Urea, mmol/L	106	35.2 [28.0; 42.5]	32	24.5 [18.0; 41.5]	0.35
LDH, U/L	101	2997.0 [2156.5; 4074]	31	3205.0 [2349.0; 4374.0]	0.42
C3, mg/dL	93	76.0 [70.0; 87.5]	27	97.0 [79.0; 105.0]	0.0001
AST, U/L	97	156.0 [98.3; 244.0]	30	134.0 [89.5; 227.0]	0.64
ALT, U/L	94	90.5 [40.0; 189.0]	30	65.3 [36.5; 117.5]	0.16
Total bilirubin, µmol/L	92	14.0 [9.6; 21.3]	28	15.6 [12.0; 22.7]	0.23
SRP, mg/L	91	14.8 [0.9; 48.9]	27	8.2 [1.0; 14.6]	0.07
D-dimers, ng/mL	80	3382 [1539; 4872]	22	3083 [2061; 4533]	0.94
SFMC, mg%	85	8.0 [6.0; 11.0]	25	6.5 [5.0; 11.0]	0.25
Fibrinogen, g/L	85	2.77 [2.22; 3.46]	25	2.49 [2.18; 3.35]	0.71
TT, s	85	19.8 [17.0; 23.9]	18	20.8 [18.4; 22.4]	0.61
PU in the acute period	92		30		
Yes	89	96.7%	30	100%	0.42
No	3	3.3%	0	0.0%	0.42
PU upon discharge	92		30		
Yes	75	81.5%	20	66.7%	0.07
No	17	18.5%	10	33.3%	0.07

Note: n—number of patients, Me [25; 75]—median and interquartile range, *p*—significance of differences, N—norm, LDH—lactate dehydrogenase, AST—aspartate aminotransferase, ALT—alanine aminotransferase, SFMC—soluble fibrin–monomer complexes, TT—thrombin time, PU—proteinuria.

## Data Availability

The data presented in this study are available on request from the corresponding author.

## Supplementary Materials

The following supporting information can be downloaded at: https://www.mdpi.com/article/10.3390/biom13111671/s1, Table S1. The standard of the classification of the severity of the disease.
