# Supplementary material for: A Moderate Decrease in ADAMTS13 Activity Correlates with the Severity of STEC-HUS
Source: Biomolecules. 2023 Nov 20;13(11):1671. doi: 10.3390/biom13111671 (PMC10669222; doi:10.3390/biom13111671)
Supplement: Supplementary file 1 [file biomolecules-13-01671-s001.zip › biomolecules-2642057-supplementary.pdf]

Table S1. The standard of the classification of the severity of the disease.

Characteristics	Severity of the course of the STEC-HUS		
	moderate condition	severe condition	critical condition
triad of TMA (MAGA, thrombocytopenia, AKI)	+	+	+
damage to the central nervous system (convulsive syndrome, sopor, stage I coma)	-	+	-
damage to the central nervous system (stage II-III coma)	-	-	+
respiratory and cardiotonic support	-	-	+
